# Distribution of metals exposure and associations with cardiometabolic risk factors in the “Modeling the Epidemiologic Transition Study”

**DOI:** 10.1186/1476-069X-13-90

**Published:** 2014-11-05

**Authors:** Adrienne S Ettinger, Pascal Bovet, Jacob Plange-Rhule, Terrence E Forrester, Estelle V Lambert, Nicola Lupoli, James Shine, Lara R Dugas, David Shoham, Ramon A Durazo-Arvizu, Richard S Cooper, Amy Luke

**Affiliations:** Yale Center for Perinatal, Pediatric & Environmental Epidemiology, Department of Chronic Disease Epidemiology, Yale School of Public Health, 1 Church St, New Haven, CT 06510 USA; Institute of Social and Preventive Medicine, Lausanne University Hospital, 10, route de la Corniche, Lausanne, CH-1010 Switzerland; Unit for the Prevention and Control of Cardiovascular Disease, Ministry of Health, Victoria, Mahe Seychelles; Kwame Nkrumah University of Science and Technology, Kumasi, Ashanti Ghana; St George’s, University of London, London, UK; UWI Solutions for Developing Countries, University of the West Indies Mona, 25 West Road, Kingston 7, Jamaica; Research Unit for Exercise Science and Sports Medicine, Division of Health Sciences, University of Cape Town, Cape Town, 7725 South Africa; Department of Environmental Health, Harvard School of Public Health, 677 Huntington Ave, Boston, MA 02115 USA; Department of Public Health Sciences, Stritch School of Medicine, Loyola University Chicago, 2160 S. First Ave, Maywood, IL 60153 USA

**Keywords:** Arsenic, Cadmium, Cardiometabolic, Diabetes, Endocrine disruption, Lead, Mercury, Metals exposure, Obesity, Risk factor

## Abstract

**Background:**

Metals are known endocrine disruptors and have been linked to cardiometabolic diseases via multiple potential mechanisms, yet few human studies have both the exposure variability and biologically-relevant phenotype data available. We sought to examine the distribution of metals exposure and potential associations with cardiometabolic risk factors in the “Modeling the Epidemiologic Transition Study” (METS), a prospective cohort study designed to assess energy balance and change in body weight, diabetes and cardiovascular disease risk in five countries at different stages of social and economic development.

**Methods:**

Young adults (25–45 years) of African descent were enrolled (N = 500 from each site) in: Ghana, South Africa, Seychelles, Jamaica and the U.S.A. We randomly selected 150 blood samples (N = 30 from each site) to determine concentrations of selected metals (arsenic, cadmium, lead, mercury) in a subset of participants at baseline and to examine associations with cardiometabolic risk factors.

**Results:**

Median (interquartile range) metal concentrations (μg/L) were: arsenic 8.5 (7.7); cadmium 0.01 (0.8); lead 16.6 (16.1); and mercury 1.5 (5.0). There were significant differences in metals concentrations by: site location, paid employment status, education, marital status, smoking, alcohol use, and fish intake. After adjusting for these covariates plus age and sex, arsenic (OR 4.1, 95% C.I. 1.2, 14.6) and lead (OR 4.0, 95% C.I. 1.6, 9.6) above the median values were significantly associated with elevated fasting glucose. These associations increased when models were further adjusted for percent body fat: arsenic (OR 5.6, 95% C.I. 1.5, 21.2) and lead (OR 5.0, 95% C.I. 2.0, 12.7). Cadmium and mercury were also related with increased odds of elevated fasting glucose, but the associations were not statistically significant. Arsenic was significantly associated with increased odds of low HDL cholesterol both with (OR 8.0, 95% C.I. 1.8, 35.0) and without (OR 5.9, 95% C.I. 1.5, 23.1) adjustment for percent body fat.

**Conclusions:**

While not consistent for all cardiometabolic disease markers, these results are suggestive of potentially important associations between metals exposure and cardiometabolic risk. Future studies will examine these associations in the larger cohort over time.

## Background

There is increasing evidence that environmental chemicals may be contributing to the risk of diabetes [1], obesity [2], and cardiovascular disease [3]. Approximately 40% of adults in the U.S. have diabetes or pre-diabetes [4] and about 70% of this risk can be attributed to obesity [5, 6] which is due, in part, to underlying genetics, nutrition, and lifestyle factors. Almost 35% of adults in the U.S. are obese [7] and, worldwide, the prevalence of obesity and of diabetes are increasing at alarming rates in industrialized and developing countries alike [8]. However, the rapid rise in obesity rates may not be explained solely by genetics and lifestyle factors. Environmental toxicants have been implicated as “obesogens” [9, 10] and risk factors for diabetes [11, 12] and cardiovascular disease [3]. Numerous questions remain, however, regarding the timing of critical windows of exposure, susceptible populations at risk, and biological mechanisms of effect.

Toxic metals (arsenic, cadmium, lead, mercury) are ubiquitous environmental contaminants that can cause a wide range of adverse health effects in humans [13, 14]. Metals have been identified as endocrine disruptors [15, 16] and, as such, potential etiologic factors with respect to diabetes. Exposure to metals may result in abnormal glucose metabolism, in turn increasing the risk of developing diabetes, through several plausible mechanisms [11], such as the induction of oxidative stress or interference in signal transduction or gene expression, resulting in beta-cell dysfunction and insulin resistance [17, 18]. Metals have also been implicated in influencing estrogen receptor-mediated signaling [19] and may disrupt the glucocorticoid receptor [20], which regulates a wide range of biological processes in humans, including insulin sensitivity. It is also possible that reverse causality is at play, i.e., the existence of diabetes or pre-diabetes alters the metabolism of metals so that higher concentrations in the body result. Experimental evidence, however, suggests that insulin resistance and oxidative stress can be induced by metals, providing biological plausibility to metals-induced diabetes [17].

Metals have also been implicated in the risk of cardiovascular disease [21] which itself is a leading cause of morbidity and mortality. The cardiovascular effects of metals are related to the increased oxidative stress and inflammation resulting from the high affinity of metals for binding sulfhydryl groups thereby reducing oxidative defense mechanisms [22]. In vivo and in vitro studies have shown that metals may exert these effects through increased thrombosis, vascular smooth muscle dysfunction, endothelial dysfunction, dyslipidemia, and immune and mitochondrial dysfunction [23]. In epidemiologic studies, metals have been associated with hypertension, impaired kidney function, and peripheral arterial disease as well as all-cause and cardiovascular mortality [24–27].

Metabolic syndrome, a clustering of physiologic abnormalities (central obesity, dyslipidemia, hyperglycemia, hypertension) and a precursor of diabetes and cardiovascular disease, has also been associated with metals exposures in cross-sectional studies [28, 29]. Thus far, however, the human evidence is limited and there is little understanding of the underlying etiology. The underlying pathophysiology connecting the different aspects of metabolic syndrome is an area of active interest and research, but it is clear that adiposity and insulin resistance play an important role [30]. Adipose tissue, as an important endocrine organ that modulates metabolism, inflammation and endothelial function, has been recognized in the development of metabolic syndrome, type 2 diabetes, and cardiovascular disease [31] and the state of obesity, itself, is an independent risk factor for cardiovascular disease [32] and type 2 diabetes [33]. However, the existence of “metabolically healthy” obese and “metabolically obese” normal weight phenotypes (31.7% of obese and 23.5% of normal weight adults in NHANES 1999–2004 [34]) indicate that there is considerable unexplained variation in these associations [35, 36] which is not explained by diet and physical activity [37]. Although, others have shown that there is no healthy pattern of increased weight [38]. Given the sheer magnitude of the obesity, type 2 diabetes, and cardiovascular disease epidemics, it is imperative to consider a potential role of non-traditional risk factors, such as environmental chemicals, to better understand their contribution to the global disease burden.

To date, it has been difficult to assess the cardiometabolic effects of environmental metals exposure in human populations given the lack of specific outcome measurements and control of potential confounding variables in existing observational data and the inherent difficulty of testing these hypotheses through clinical trials. Many studies have focused on body mass index (BMI) and there is little information on the biologically-relevant phenotypes of obesity (e.g. fat distribution, fat/lean mass percentages) or the mechanistic intermediate markers of metabolic syndrome.

The objectives of this study were to: i) examine the distribution of selected blood metals (arsenic, cadmium, lead, mercury) and ii) evaluate the independent associations of blood metals with clinical markers of cardiometabolic risk in a cohort of young adults of African descent living in five countries across a range of social and economic development. In addition to the extensive phenotype data and stored biological specimens already available on this large sample population, a major strength is the widespread geographic distribution of the study sites which may underlie largely different exposures across the sites.

## Methods

### Study population

The “Modeling the Epidemiologic Transition Study” (METS) is a prospective cohort study designed to assess the association between physical activity levels and relative weight, weight gain and diabetes and cardiovascular disease risk in five population-based samples at different stages of social and economic development [39]. Twenty-five hundred (2,500) young adults, age 25–45 years, were enrolled between January 2010 and September 2011 with 500 participants (~50% male) from each of five sites: rural Ghana (Kumasi), urban South Africa (Cape Town), the island nation of Seychelles (Victoria), urban Jamaica (Kingston) and the suburban Chicago area (Maywood, IL) of the United States of America (U.S.A.). The populations sampled are all of African descent and represent a range of social and economic development as defined by the United Nations Human Development Index (HDI) 2011 [40]: Ghana (HDI = 0.541) as a lower “medium” HDI country, South Africa (0.619) as “medium”, Jamaica (0.727) and Seychelles (0.773) as “high”, and the U.S.A. (0.910) as “very high”. The present study includes a subset of 150 individuals from the cohort with metals concentrations measured in randomly selected baseline blood samples.

Extensive baseline data collection included: anthropometrics, estimates of body composition by bioelectrical impedance analysis, blood pressure, physical activity/inactivity by accelerometry and the World Health Organization Global Physical Activity Questionnaire [41, 42], dietary intake by two 24-hour recalls, smoking and alcohol consumption history, self- and family medical history, and measures of employment history, occupation, education and wealth using the World Bank’s Core Welfare Indicators Questionnaire [43]. Fasting blood samples were collected and analyzed for glucose, lipids, C-reactive protein, and cystatin C. Yearly follow-up examinations include measurement of body weight, blood pressure and change in medical history. Participants have been examined annually for the past two-to-three years and will continue to be followed for assessment of change in body weight and composition and diabetes and cardiovascular disease risk. Exclusion criteria for METS included: individuals with obvious infectious diseases (including active malaria), HIV-positive individuals, pregnant or lactating women, and individuals with conditions which prevented them from engaging in normal day-to-day physical activities (such as severe osteo- or rheumatoid arthritis or lower extremity disability). The research protocol was approved by the Human Subjects Committees of the participating institutions.

### Exposure assessment

We randomly selected 150 baseline samples from archived whole blood specimens (n = 30 per site) to be measured for concentrations of arsenic, cadmium, lead, and mercury. Venous blood samples were collected in 6-ml capacity plastic lavender top hematology Vacutainer™ tubes with EDTA as an anticoagulant (BD #367863, Becton Dickinson, Franklin Lakes, NJ) and mixed by inverting gently five times. Whole blood (0.5 ml) was transferred to 2-ml polypropylene microcentrifuge tubes with screw caps (Fisherbrand™ #02-682-558, Fisher Scientific Company L.L.C., Pittsburgh, PA), stored at -80°C. Samples were shipped on dry ice to the Harvard School of Public Health Trace Metals Laboratory (Boston, MA) for analysis.

All samples were handled in a Class 10,000 clean room under a Class 100 clean hood. All glass and plastic ware were cleaned by soaking in 10% HNO_3_ for 24 hours and rinsing several times with deionized water. Blood samples were weighed and digested in 1 ml of ultrapure concentrated HNO_3_ acid for 24 hours and then diluted to 5 ml with deionized water after the addition of 0.5 ml of 30% ultrapure hydrogen peroxide. Samples were analyzed using a dynamic reaction cell-inductively coupled plasma mass spectrometer (DRC-ICP-MS, Elan 6100, Perkin Elmer, Norwalk, CT). A mixed solution of 50 ng/ml In (for cadmium), 50 ng/ml Lu (for lead) and 150 ng/ml Te (for arsenic) in 5% HNO_3_ were used as the internal standards. This solution was mixed with calibration standards and samples on-line using a mixing tee and a mixing coil. Samples were analyzed by the external calibration method using seven standards with concentrations ranging from 0 to 10 ng/ml. Quality control measures included: analysis of initial calibration verification standard, continuous calibration standard, procedural blanks, duplicate samples, spiked samples, National Institute of Standard and Technology Standard Reference Material (NIST SRM) 1643e-trace elements in water and NIST SRM955b-lead in blood. Results given were the average of five replicate measurements. The limits of detection (ng/ml) in blood for this procedure are: arsenic (0.02); cadmium (0.01); and lead (0.01). Total mercury was analyzed using the Direct Mercury Analyzer 80 Tricell (Milestone Inc., Monroe, CT) with a detection limit equal to 0.0015 ng/ml.

### Statistical analysis

Participant characteristics were summarized by sex: overall and by site. Blood metals concentrations were summarized overall and by site for each metal and by participant characteristics. For metals levels less than the limit of detection (LOD), a random number between 0 and LOD was substituted according to a uniform distribution. Next, metals concentrations were summarized for subjects with and without each of 11 recognized cardiometabolic disease risk factors dichotomized using standard clinical cut-points. Metabolic syndrome is diagnosed based on abnormalities in specific clustered clinical cardiometabolic measures that are associated with increased risk for diabetes and cardiovascular disease [44]. However, there are no specific cutoffs for populations of African descent, so we used cutoffs values for Europeans as suggested by the International Diabetes Federation [45]. Logistic regression was used to describe associations between metals exposure (dichotomized at the median) and cardiometabolic risk factors and odds ratios (OR) with 95% confidence intervals (CI) presented. The main covariates of interest were determined *a priori* based on biological considerations (age, sex, smoking, alcohol use, fish intake, percent body fat, physical activity) and others (education, marital status, paid employment status, site location) included using statistical considerations if they were significant (p = 0.1) in bivariate models.

Final models were adjusted for “core” variables including: age, sex, and site location. “Fully-adjusted” models additionally included: education, paid employment, marital status smoking, alcohol use, and fish intake. Since adiposity may be a mediator, modifier, or outcome as a result of exposure to environmental chemicals, we did not adjust our initial models for BMI or other markers of excess body fat. However, we next additionally adjusted “core” and “fully-adjusted” models for percent body fat. All analyses were performed using STATA® version 11.2 (Copyright 1985–2009, StataCorp LP College Station, Texas USA).

## Results

As a result of the parent (METS) study design, participant characteristics varied widely across sites (Table 1). Since baseline blood samples (N = 30 per site) were chosen randomly, the distribution of participants by sex within each site is not uniform; however, overall there were 74 males (49%) and 76 females (51%) participants included with mean (±SD) age of 34.7 (±6.0) and 35.2 (±6.2) years, respectively. Forty-nine percent (N = 74) of participants were classified as overweight and 29% (N = 44) as obese by BMI; similarly, 49% had signs of abdominal adiposity measured by sex-specific elevated waist circumference.

**Table 1:** **Distribution of participant characteristics by sex: overall and by site location**

	Overall	U.S.A.	South Africa	Ghana	Jamaica	Seychelles	
	Mean ± SD (or %)	Mean ± SD (or %)	Mean ± SD (or %)	Mean ± SD (or %)	Mean ± SD (or %)	Mean ± SD (or %)	p-value*
**Male**	**N = 74**	**N = 10**	**N = 15**	**N = 10**	**N = 24**	**N = 15**	
Age (years)	34.7 ± 6.0	34.8 ± 6.4	33.7 ± 6.0	36.5 ± 6.4	34.5 ± 6.4	34.6 ± 5.3	0.9
Education (years)	11.2 ± 2.2	12.7 ± 1.4	10.3 ± 1.9	9.2 ± 2.4	11.1 ± 1.9	12.8 ± 1.7	<0.0001
Weight (kg)	74.0 ± 17.2	89.1 ± 20.2	64.1 ± 16.4	59.3 ± 6.0	75.4 ± 12.9	81.2 ± 15.3	<0.0001
Height (cm)	172.8 ± 7.4	171.5 ± 4.3	166.4 ± 6.1	170.3 ± 6.4	177.7 ± 6.5	173.9 ± 7.2	<0.0001
Body Mass Index (kg/m^2^)	24.7 ± 5.4	30.4 ± 7.2	22.9 ± 4.5	20.5 ± 2.2	23.9 ± 4.1	26.9 ± 4.9	<0.0001
Waist circumference (cm)	83.2 ± 13.5	93.5 ± 20.6	79.3 ± 12.3	74.2 ± 5.4	81.8 ± 10.6	88.8 ± 11.5	0.004
Fat Mass (kg)	20.2 ± 11.1	31.5 ± 13.6	18.7 ± 9.7	10.1 ± 5.7	19.4 ± 8.5	22.4 ± 10.9	<0.0001
Percent Body Fat (%)	25.9 ± 9.2	34.0 ± 7.8	27.7 ± 8.1	16.8 ± 8.9	24.8 ± 8.2	26.4 ± 8.0	<0.0001
Overweight (BMI ≥25)	40.5	70.0	26.7	10.0	37.5	60.0	0.02
Obese (BMI ≥30)	14.9	60.0	6.7	0.0	4.2	20.0	<0.0001
Employed	74.3	60.0	26.7	90.0	87.5	100.0	<0.0001
Married (or Living as Married)	51.4	40.0	40.0	90.0	37.5	66.7	0.03
Smoking (ever)	37.8	40.0	66.7	0.0	33.3	40.0	0.02
Alcohol (≥12 drinks/year)	68.9	60.0	40.0	40.0	83.3	100.0	<0.0001
Fish Intake (≥1 serving/day)	55.4	20.0	20.0	80.0	62.5	86.7	0.0001
Physical Activity (<21.5 min/day)	60.8	80.0	33.3	50.0	70.8	66.7	0.09
**Female**	**N = 76**	**N = 20**	**N = 15**	**N = 20**	**N = 6**	**N = 15**	
Age (years)	35.2 ± 6.2	37.1 ± 5.9	33.7 ± 6.8	33.2 ± 6.5	37.2 ± 4.6	36.0 ± 5.8	0.2
Education (years)	11.0 ± 4.3	14.1 ± 2.4	9.7 ± 3.4	6.9 ± 3.5	10.3 ± 1.8	14.0 ± 3.7	<0.0001
Weight (kg)	74.8 ± 20.5	77.7 ± 19.0	81.6 ± 24.3	67.8 ± 16.1	91.2 ± 32.2	67.1 ± 12.3	0.03
Height (cm)	161.5 ± 7.5	164.3 ± 8.8	160.1 ± 5.9	157.5 ± 7.2	165.8 ± 8.3	162.8 ± 4.8	0.02
Body Mass Index (kg/m^2^)	28.7 ± 7.5	28.6 ± 5.3	31.9 ± 9.5	27.3 ± 6.5	33.3 ± 12.1	25.4 ± 5.2	0.07
Waist circumference (cm)	90.4 ± 14.9	90.9 ± 14.7	95.7 ± 15.3	88.5 ± 13.3	101.1 ± 21.2	82.6 ± 10.2	0.05
Fat Mass (kg)	30.5 ± 13.8	32.3 ± 11.5	37.7 ± 15.5	25.3 ± 12.0	39.0 ± 21.7	24.6 ± 8.5	0.01
Percent Body Fat (%)	39.4 ± 8.7	40.9 ± 6.8	45.1 ± 6.9	35.9 ± 10.3	40.4 ± 10.3	35.8 ± 6.8	0.009
Overweight (BMI ≥25)	57.9	50.0	80.0	45.0	83.3	53.3	0.2
Obese (BMI ≥30)	43.4	50.0	46.7	45.0	50.0	26.7	0.7
Employed	52.6	60.0	6.7	50.0	33.3	100.0	<0.0001
Married (or Living as Married)	47.4	20.0	53.3	65.0	50.0	53.3	0.06
Smoking (ever)	15.8	40.0	6.7	0.0	33.3	6.7	0.002
Alcohol (≥12 drinks/year)	42.1	55.0	20.0	35.0	66.7	46.7	0.2
Fish Intake (≥1 serving/day)	51.3	40.0	13.3	75.0	33.3	80.0	0.0002
Physical Activity (<21.5 min/day)	84.2	90.0	93.3	80.0	83.3	73.3	0.6

*p-value from analysis of variation by site.

Median (interquartile range) metals concentrations (μg/L) were: arsenic 8.5 (7.7); cadmium 0.01 (0.8); lead 16.6 (16.1); and mercury 1.5 (5.0). Overall, metals were significantly correlated with each other, for example: arsenic was significantly correlated with: lead (ρ = 0.3, p < 0.001), cadmium (ρ = 0.5, p < 0.00001), and mercury (ρ = 0.6, p < 0.00001). However, the strengths of these correlations varied between sites with the highest correlations among the metals observed in Jamaica and the lowest correlations among the metals in the U.S. (data not shown).

Overall, almost 63% and over 15% of samples, respectively, for cadmium and mercury were below the limits of detection (Table 2). No samples had undetectable levels of arsenic and less than one percent had undetectable levels of lead. As expected, metals concentrations differed significantly by site location. Ghana had the highest median blood lead levels and the Seychelles had the highest median blood levels of arsenic and mercury. Twenty-three percent (N = 7) of Ghanaian participants, representing 20% (N = 2) and 25% (N = 5) of men and women, respectively, had blood lead levels >10 μg/dL and one (3%) female participant had a blood lead level >25 μg/dL. Forty-three percent of Ghanaians had blood lead levels >3.6 μg/dL (95th percentile of NHANES 2009–2010 levels for ages 20 years and above) [46] and 17% of Seychellois were also above this threshold. Interestingly, one participant at each site in the U.S.A. and South Africa also exceeded the NHANES 95th percentile for blood lead while none in Jamaica did.

**Table 2:** **METS blood metals concentrations, overall and by site location**

						Selected percentiles						
	N	GM	(95% CI)	% < LOD*	Minimum**	25th	(95% CI)	50th	(95% CI)	75th	(95% CI)	Maximum
**Overall**												
Lead (μg/dL)	150	1.55	(1.30, 1.85)	0.7	0.001	0.97	(0.76, 1.09)	1.66	(1.34, 1.93)	2.60	(2.25, 2.98)	31.82
Cadmium (μg/L)	150	0.03	(0.02, 0.04)	62.7	0.000004	0.004	(0.003, 0.005)	0.008	(0.007, 0.009)	0.83	(0.29, 1.08)	3.08
Arsenic (μg/L)	150	8.23	(7.50, 9.02)	0.0	2.51	4.92	(4.57, 5.87)	8.48	(7.62, 10.07)	12.76	(11.59, 14.99)	26.85
Mercury (μg/L)	150	0.85	(0.52, 1.37)	15.3	0.001	0.22	(0.07, 0.51)	1.49	(1.01, 2.23)	5.31	(3.42, 15.38)	70.36
**U.S.A.**												
Lead (μg/dL)	30	0.80	(0.61, 1.05)	0.0	0.22	0.44	(0.36, 0.68)	0.74	(0.47, 1.14)	1.28	(0.95, 1.89)	6.05
Cadmium (μg/L)	30	0.15	(0.07, 0.31)	26.7	0.005	0.009	(0.006, 0.20)	0.29	(0.14, 0.60)	0.83	(0.36, 1.03)	1.21
Arsenic (μg/L)	30	3.97	(3.62, 4.35)	0.0	2.61	3.33	(3.02, 3.55)	3.83	(3.42, 4.56)	4.72	(3.94, 5.24)	8.47
Mercury (μg/L)	30	0.07	(0.03, 0.20)	33.3	0.001	0.007	(0.002, 0.07)	0.15	(0.01, 0.36)	0.69	(0.22, 1.22)	24.46
**South Africa**												
Lead (μg/dL)	30	1.45	(1.22, 1.74)	0.0	0.58	0.99	(0.82, 1.23)	1.42	(1.16, 1.86)	1.99	(1.71, 2.74)	3.79
Cadmium (μg/L)	30	0.00	(0.00, 0.01)	93.3	0.000004	0.002	(0.0006, 0.003)	0.004	(0.003, 0.007)	0.008	(0.005, 0.01)	1.10
Arsenic (μg/L)	30	5.65	(5.18, 6.16)	0.0	3.98	4.71	(4.32, 5.11)	5.43	(4.81, 6.52)	6.88	(5.84, 7.71)	9.52
Mercury (μg/L)	30	0.05	(0.02, 0.15)	40.0	0.001	0.005	(0.001, 0.03)	0.09	(0.01, 0.33)	0.47	(0.15, 0.97)	35.10
**Ghana**												
Lead (μg/dL)	30	3.12	(1.53, 6.38)	3.3	0.001	2.02	(1.35, 2.79)	2.96	(2.14, 3.96)	5.90	(3.59, 25.97)	31.82
Cadmium (μg/L)	30	0.00	(0.00, 0.01)	100.0	0.00003	0.004	(0.001, 0.005)	0.006	(0.004, 0.007)	0.008	(0.006, 0.009)	0.01
Arsenic (μg/L)	30	11.17	(9.69, 12.87)	0.0	2.51	10.30	(7.47, 11.11)	11.68	(10.46, 12.57)	14.00	(12.31, 15.61)	20.70
Mercury (μg/L)	30	1.56	(1.01, 2.42)	3.3	0.009	1.08	(0.73, 1.42)	1.87	(1.33, 2.29)	2.93	(2.14, 4.13)	11.25
**Jamaica**												
Lead (μg/dL)	30	0.96	(0.77, 1.19)	0.0	0.25	0.69	(0.44, 0.95)	1.01	(0.77, 1.24)	1.39	(1.06, 2.01)	2.57
Cadmium (μg/L)	30	0.01	(0.00, 0.04)	76.7	0.0002	0.004	(0.001, 0.004)	0.005	(0.00, 0.01)	0.17	(0.01, 1.10)	3.08
Arsenic (μg/L)	30	9.24	(8.37, 10.19)	0.0	6.35	7.89	(6.99, 8.49)	8.61	(8.09, 9.65)	10.23	(8.97, 14.20)	19.02
Mercury (μg/L)	30	2.43	(1.82, 3.25)	0.0	0.22	1.66	(0.82, 2.40)	2.54	(2.19, 3.60)	3.84	(3.39, 6.41)	8.33
**Seychelles**												
Lead (μg/dL)	30	2.56	(2.26, 2.90)	0.0	1.46	1.96	(1.72, 2.43)	2.49	(2.21, 2.80)	3.23	(2.60, 3.92)	6.71
Cadmium (μg/L)	30	0.47	(0.21, 1.06)	16.7	0.001	1.04	(0.01, 1.15)	1.17	(1.11, 1.22)	1.27	(1.19, 1.42)	1.56
Arsenic (μg/L)	30	16.29	(14.93, 17.77)	0.0	10.55	13.98	(11.93, 15.56)	16.38	(14.40, 17.05)	18.59	(16.59, 23.10)	26.85
Mercury (μg/L)	30	28.47	(23.03, 35.18)	0.0	9.20	18.16	(13.70, 24.45)	28.34	(22.34, 39.19)	48.58	(31.77, 60.84)	70.36

*Limits of detection (ng/ml): Lead (0.01); Cadmium (0.01); Arsenic (0.02); Mercury (0.0015).

**For levels less than LOD: substituted a random number between 0 and LOD (according to uniform distribution).

There were no significant differences in blood metals by age group or sex, although in both the U.S. and South African samples women tended to have lower lead levels than the men. There were also significant differences in metals concentrations by: employment status, education, marital status, smoking, alcohol use, and fish intake (Table 3). Those participants reporting paid employment in the previous month had higher blood metals concentrations than those who did not work and these differences were significantly significant for all of the metals, except lead, suggesting that occupation may be related to exposure. However, there were no significant differences by employment type: non-manual, skilled manual, or unskilled manual labor. Most participants (73%) in this subsample of the cohort reported being “never smokers” and only 18% (N = 27) reported being “current” (≥1/day for at least one year) smokers. Among smokers, the mean (±SD) number of cigarettes smoked per day was 6.9 ± 4.2 (range 1–20) and this did not differ significantly by location (p = 0.84). As would be expected, cadmium levels were significantly higher among current or occasional smokers compared to ex- or never smokers (p = 0.008).

**Table 3:** **Geometric mean (95% confidence interval) blood metals concentrations by participant characteristics**

		Lead (μg/dL)		Cadmium (μg/L)		Arsenic (μg/L)		Mercury (μg/L)	
Variable	N (%)	GM (95% CI)	p-value	GM (95% CI)	p-value	GM (95% CI)	p-value	GM (95% CI)	p-value
**Site Location**									
U.S.A.	30 (20)	0.80 (0.61, 1.05)	<0.00001	0.15 (0.07, 0.31)	<0.00001	3.97 (3.62, 4.35)	<0.00001	0.07 (0.03, 0.20)	<0.00001
South Africa	30 (20)	1.45 (1.22, 1.74)		0.00 (0.00, 0.01)		5.65 (5.18, 6.16)		0.05 (0.02, 0.15)	
Ghana	30 (20)	3.12 (1.53, 6.38)		0.00 (0.00, 0.01)		11.17 (9.69, 12.87)		1.56 (1.01, 2.42)	
Jamaica	30 (20)	0.96 (0.77, 1.19)		0.01 (0.00, 0.04)		9.24 (8.37, 10.19)		2.43 (1.82, 3.25)	
Seychelles	30 (20)	2.56 (2.26, 2.90)		0.47 (0.21, 1.06)		16.29 (14.93, 17.77)		28.47 (23.03, 35.18)	
**Age group**									
<35 years	72 (48)	1.46 (1.07, 1.99)	0.64	0.02 (0.01, 0.04)	0.09	8.13 (7.17, 9.22)	0.53	0.81 (0.41, 1.61)	0.22
≥35 years	78 (52)	1.64 (1.35, 1.99)		0.04 (0.02, 0.07)		8.32 (7.26, 9.53)		0.88 (0.44, 1.75)	
**Sex**									
Male	74 (49)	1.68 (1.41, 2.00)	0.35	0.03 (0.02, 0.06)	0.43	8.94 (7.93, 10.07)	0.21	1.61 (0.88, 2.94)	0.13
Female	76 (51)	1.44 (1.05, 1.96)		0.02 (0.01, 0.05)		7.59 (6.59, 8.73)		0.45 (0.22, 0.94)	
**Marital Status**									
Married/living as married	74 (49)	1.97 (1.57, 2.48)	0.02	0.02 (0.01, 0.04)	0.38	9.48 (8.39, 10.71)	0.007	1.40 (0.74, 2.65)	0.08
Not married*	76 (51)	1.22 (0.94, 1.60)		0.03 (0.02, 0.06)		7.17 (6.28, 8.18)		0.52 (0.25, 1.06)	
**Education**									
≤11 years	83 (55)	1.84 (1.39, 2.44)	0.03	0.01 (0.01, 0.02)	0.03	9.09 (8.20, 10.09)	0.23	0.90 (0.51, 1.60)	0.004
>11 years	67 (45)	1.25 (1.03, 1.52)		0.07 (0.04, 0.14)		7.27 (6.20, 8.52)		0.78 (0.35, 1.78)	
**Paid Employment (past month)**									
Yes	95 (63)	1.71 (1.42, 2.06)	0.81	0.05 (0.03, 0.09)	<0.00001	9.63 (8.60, 10.79)	<0.00001	2.24 (1.33, 3.80)	<0.00001
No	55 (37)	1.31 (0.90, 1.90)		0.01 (0.00, 0.02)		6.26 (5.48, 7.15)		0.16 (0.07, 0.34)	
**Employment type**									
Non-manual labor	37 (25)	1.07 (0.78, 1.45)	0.12	0.10 (0.04, 0.22)	0.22	7.18 (5.67, 9.09)	0.38	0.76 (0.25, 2.32)	0.22
Skilled manual labor	16 (11)	1.39 (1.03, 1.88)		0.03 (0.01, 0.13)		7.73 (6.08, 9.81)		0.63 (0.13, 3.12)	
Unskilled manual labor	92 (61)	1.87 (1.45, 2.41)		0.02 (0.01, 0.03)		9.02 (8.12, 10.03)		1.10 (0.63, 1.93)	
Missing	5 (3)	1.01 (0.45, 2.31)		0.02 (0.00, 0.35)		5.01 (2.77, 9.09)		0.04 (0.00, 2.76)	
**Cigarette smoking**									
Smoker (≥1/day for year)	27 (18)	1.58 (1.28, 1.96)	0.67	0.13 (0.04, 0.37)	0.0008	6.34 (5.12, 7.85)	0.03	0.20 (0.06, 0.74)	0.05
Occasional smoker	8 (5)	2.22 (1.59, 3.10)		0.05 (0.00, 1.30)		12.09 (8.03, 18.20)		5.96 (0.56, 63.89)	
Ex-smoker (for at least 1 year)	5 (3)	1.61 (0.61, 4.26)		0.01 (0.00, 0.27)		8.79 (5.68, 13.60)		2.21 (0.23, 21.52)	
Never-smoker	110 (73)	1.50 (1.18, 1.90)		0.02 (0.01, 0.03)		8.50 (7.63, 9.47)		1.00 (0.59, 1.71)	
**Alcohol Use (≥12 drinks/year)**									
Yes	83 (55)	1.42 (1.10, 1.84)	0.06	0.05 (0.03, 0.09)	0.002	8.94 (7.88, 10.15)	0.031	1.59 (0.84, 3.01)	0.006
No/Missing	67 (45)	1.72 (1.34, 2.22)		0.01 (0.01, 0.02)		7.41 (6.49, 8.47)		0.39 (0.19, 0.78)	
**Fish Intake (≥1 serving/day)**									
Yes	80 (53.3)	2.13 (1.72, 2.65)	0.003	0.46 (0.02, 0.09)	0.0009	10.59 (9.40, 11.93)	<0.00001	2.85 (1.72, 4.72)	0.0002
No	70 (46.7)	1.07 (0.82, 1.41)		0.01 (0.01, 0.03)		6.16 (5.52, 6.88)		0.21 (0.10, 0.44)	
**Physical Activity**									
<21.5 mins/day	109 (73)	1.39 (1.11, 1.75)	0.62	0.03 (0.02, 0.05)	0.98	7.87 (7.02, 8.82)	0.36	0.79 (0.44, 1.41)	0.68
≥21.5 mins/day	41 (27)	2.06 (1.61, 2.64)		0.03 (0.01, 0.06)		9.25 (7.98, 10.73)		1.02 (0.44, 2.40)	
**Weight group**									
Underweight, BMI < 18.5	6 (4)	1.83 (1.21, 2.77)	0.61	0.00 (0.00, 0.01)	0.47	8.06 (5.02, 12.92)	0.11	0.39 (0.00, 31.81)	0.04
Normal weight, BMI 18.5 to <25	70 (47)	1.62 (1.17, 2.26)		0.03 (0.02, 0.06)		8.74 (7.62, 10.01)		0.91 (0.48, 1.75)	
Overweight, BMI ≥25 to <30	30 (20)	1.67 (1.24, 2.24)		0.01 (0.00, 0.05)		9.55 (7.91, 11.53)		2.38 (0.86, 6.59)	
Obese, BMI ≥30	44 (29)	1.34 (1.04, 1.73)		0.04 (0.02, 0.09)		6.77 (5.66, 8.10)		0.41 (0.16, 1.09)	

*Not married includes: widowed, divorced, separated, and never married.

Table 4 shows unadjusted associations between metals and cardiometabolic risk factors. Less than 3% and 7% of participants in the sample, respectively, reported having diabetes or high blood pressure. However, 34% (N = 51) had elevated fasting blood glucose levels and 16% (N = 24) had elevated systolic and 15.3% (N = 23) had elevated diastolic blood pressure by baseline clinical measurement. Additionally, 30% (N = 45) had elevated triglyceride levels and more than 31% (N = 47) had low HDL cholesterol while more than 67% (N = 101) had elevated LDL cholesterol. Geometric mean (95% CI) metals levels were higher in those participants with elevated fasting glucose. Arsenic and mercury levels were also significantly higher in those with elevated triglyceride to HDL ratios.

**Table 4:** **Unadjusted associations between metals exposures and cardiometabolic risk factors**

		Lead (μg/dL)		Cadmium (μg/L)		Arsenic (μg/L)		Mercury (μg/L)	
Cardiometabolic risk factor	N (%)	GM (95% CI)	p-value	GM (95% CI)	p-value	GM (95% CI)	p-value	GM (95% CI)	p-value
Overweight, BMI ≥25									
Yes	74 (49.3)	1.46 (1.21, 1.77)	0.53	0.03 (0.01, 0.05)	0.84	7.78 (6.80, 8.90)	0.24	0.84 (0.41, 1.73)	0.98
No	76 (50.7)	1.64 (1.21, 2.22)		0.03 (0.01, 0.05)		8.68 (7.64, 9.87)		0.85 (0.44, 1.64)	
Obese, BMI ≥30									
Yes	44 (29.3)	1.34 (1.04, 1.73)	0.31	0.04 (0.02, 0.09)	0.18	6.77 (5.66, 8.10)	0.008	0.41 (0.16, 1.09)	0.06
No	106 (70.7)	1.65 (1.31, 2.08)		0.02 (0.01, 0.04)		8.92 (8.03, 9.90)		1.14 (0.66, 1.97)	
Waist Circumference ≥94 cm (males) or ≥80 cm (females)									
Yes	74 (49.3)	1.49 (1.21, 1.84)	0.69	0.03 (0.02, 0.07)	0.31	7.35 (6.40, 8.44)	0.02	0.55 (0.26, 1.18)	0.08
No	76 (50.7)	1.61 (1.20, 2.15)		0.02 (0.01, 0.04)		9.18 (8.15, 10.34)		1.29 (0.72, 2.32)	
Glucose ≥100 mg/dL (fasting)									
Yes	51 (34.0)	2.30 (1.78, 2.98)	0.002	0.06 (0.03, 0.14)	0.009	9.70 (8.19, 11.48)	0.01	1.62 (0.70, 3.76)	0.06
No	99 (66.0)	1.26 (1.01, 1.59)		0.02 (0.01, 0.03)		7.56 (6.79, 8.41)		0.61 (0.34, 1.08)	
LDL cholesterol ≥2.59 mmol/L									
Yes	101 (67.3)	1.51 (1.28, 1.77)	0.66	0.03 (0.02, 0.06)	0.11	8.42 (7.56, 9.38)	0.47	1.25 (0.71, 2.21)	0.02
No	49 (32.7)	1.64 (1.05, 2.56)		0.02 (0.01, 0.03)		7.84 (6.56, 9.36)		0.38 (0.16, 0.90)	
Triglycerides ≥1.7 mmol/L									
Yes	45 (30.0)	1.14 (0.90, 1.46)	0.05	0.02 (0.01, 0.04)	0.37	8.43 (7.33, 9.68)	0.73	1.13 (0.59, 2.17)	0.44
No	105 (70.0)	1.76 (1.40, 2.23)		0.03 (0.02, 0.05)		8.14 (7.23, 9.17)		0.75 (0.40, 1.41)	
HDL cholesterol <1.03 (males) or <1.29 (females) mmol/L									
Yes	47 (31.3)	1.69 (1.06, 2.71)	0.51	0.01 (0.01, 0.03)	0.06	8.27 (6.83, 10.00)	0.94	0.39 (0.16, 0.97)	0.04
No	103 (68.7)	1.49 (1.27, 1.74)		0.04 (0.02, 0.06)		8.21 (7.39, 9.11)		1.20 (0.69, 2.10)	
Triglyceride/HDL ratio >3.5 (males) or >2.5 (females)									
Yes	55 (36.7)	1.41 (1.17, 1.71)	0.44	0.04 (0.02, 0.08)	0.27	10.69 (9.55, 11.97)	<0.00001	4.49 (2.78, 7.26)	<0.00001
No	95 (63.3)	1.64 (1.26, 2.13)		0.02 (0.01, 0.04)		7.07 (6.26, 7.97)		0.32 (0.17, 0.61)	
C-reactive protein ≥10 mg/L									
Yes	43 (28.7)	1.14 (0.93, 1.40)	0.06	0.01 (0.01, 0.03)	0.03	8.79 (7.78, 9.92)	0.37	1.30 (0.66, 2.54)	0.27
No	107 (71.3)	1.75 (1.38, 2.21)		0.04 (0.02, 0.06)		8.01 (7.10, 9.03)		0.71 (0.38, 1.32)	
Systolic ≥130 mmHg									
Yes	24 (16.0)	1.56 (1.24, 1.96)	0.97	0.04 (0.01, 0.16)	0.40	7.08 (5.44, 9.21)	0.16	0.76 (0.22, 2.64)	0.85
No	126 (84.0)	1.55 (1.25, 1.91)		0.02 (0.02, 0.04)		8.46 (7.67, 9.34)		0.86 (0.51, 1.46)	
Diastolic ≥85 mmHg									
Yes	23 (15.3)	1.69 (1.23, 2.32)	0.68	0.03 (0.01, 0.14)	0.63	6.26 (4.80, 8.16)	0.02	0.49 (0.14, 1.77)	0.35
No	127 (84.7)	1.53 (1.24, 1.87)		0.03 (0.02, 0.04)		8.64 (7.85, 9.52)		0.93 (0.55, 1.57)	

Adjusting for age, sex, and site location, arsenic (OR 3.0, 95% C.I. 1.0, 8.6), cadmium (OR 2.1, 95% C.I. 1.0, 4.2), lead (OR 3.3, 95% C.I. 1.5, 7.1), and mercury (OR 2.0, 95% C.I. 0.7, 5.7) were associated with an increased odds of elevated fasting glucose (Table 5); these associations were stronger upon adjustment for additional covariates and including percent body fat (Table 6). Additionally, arsenic above the median value was associated with significantly increased odds low HDL cholesterol (OR 1.22, 95% C.I. 1.07, 1.38). Mercury above the median was associated with increased odds for 10 of the 11 cardiometabolic risk factors, but none of these associations was statistically significant. While these results, and several other non-significant associations, are in the hypothesized direction of increased risk, there were also some statistically significant (and non-significant) negative associations that do not support our hypotheses.

**Table 5:** Adjusted* associations between metals (dichotomized at median) and cardiometabolic risk factors

		Lead (>1.66 μg/dL)		Cadmium (>0.008 μg/L)		Arsenic (>8.48 μg/L)		Mercury (>1.49 μg/L)	
		Core model	Fully-adjusted	Core model	Fully-adjusted	Core model	Fully-adjusted	Core model	Fully-adjusted
Cardiometabolic risk factor	N (%)	OR (95% CI)*	OR (95% CI)**	OR (95% CI)*	OR (95% CI)**	OR (95% CI)*	OR (95% CI)**	OR (95% CI)*	OR (95% CI)**
Overweight, BMI ≥25	74 (49.3)	0.51 (0.24, 1.08)	0.60 (0.27, 1.36)	0.84 (0.43, 1.64)	0.71 (0.33, 1.52)	0.27 (0.10, 0.77)	0.27 (0.09, 0.81)	1.68 (0.61, 4.63)	1.73 (0.61, 4.91)
Obese, BMI ≥30	44 (29.3)	1.06 (0.45, 2.51)	1.54 (0.58, 4.11)	1.81 (0.83, 3.94)	1.89 (0.78, 4.61)	0.76 (0.24, 2.38)	0.86 (0.24, 3.07)	1.98 (0.61, 6.40)	1.98 (0.58, 6.74)
Waist Circumference ≥94 (males), ≥80 (females) cm	74 (49.3)	0.87 (0.37, 2.09)	1.40 (0.51, 3.83)	1.61 (0.72, 3.62)	2.00 (0.76, 5.27)	0.47 (0.15, 1.50)	0.54 (0.15, 1.95)	1.51 (0.46, 4.99)	1.47 (0.43, 5.00)
Glucose ≥100 mg/dL (fasting)	51 (34.0)	3.25 (1.48, 7.12)	3.95 (1.63, 9.58)	2.08 (1.03, 4.21)	1.69 (0.77, 3.68)	2.97 (1.03, 8.57)	4.10 (1.15, 14.59)	1.96 (0.68, 5.67)	1.96 (0.64, 5.98)
LDL cholesterol ≥2.59 mmol/L	101 (67.3)	0.64 (0.29, 1.39)	0.68 (0.29, 1.60)	1.38 (0.67, 2.82)	0.86 (0.37, 1.97)	0.46 (0.16, 1.36)	0.39 (0.12, 1.28)	2.09 (0.73, 5.99)	1.76 (0.59, 5.18)
Triglycerides ≥1.7 mmol/L	45 (30.0)	0.12 (0.05, 0.31)	0.09 (0.03, 0.25)	0.40 (0.18, 0.86)	0.39 (0.16, 0.95)	0.57 (0.20, 1.63)	0.52 (0.17, 1.61)	1.34 (0.45, 4.02)	1.17 (0.36, 3.77)
HDL cholesterol <1.03 (males), <1.29 (females) mmol/L	47 (31.3)	2.36 (1.00, 5.58)	1.93 (0.74, 5.02)	0.62 (0.30, 1.31)	0.64 (0.28, 1.50)	7.15 (1.99, 25.70)	5.85 (1.48, 23.07)	0.45 (0.14, 1.43)	0.51 (0.16, 1.65)
Triglyceride/HDL ratio >3.5 (males), >2.5 (females)	55 (36.7)	0.14 (0.05, 0.44)	0.14 (0.04, 0.48)	0.43 (0.16, 1.15)	0.53 (0.18, 1.55)	0.49 (0.16, 1.52)	0.56 (0.16, 1.94)	1.28 (0.39, 4.25)	1.15 (0.31, 4.32)
C-reactive protein ≥10 mg/L	43 (28.7)	0.15 (0.06, 0.39)	0.13 (0.04, 0.36)	0.20 (0.08, 0.49)	0.28 (0.10, 0.75)	0.38 (0.13, 1.12)	0.45 (0.14, 1.44)	1.21 (0.39, 3.74)	1.14 (0.33, 3.98)
Systolic ≥130 mmHg	24 (16.0)	1.50 (0.56, 4.03)	1.69 (0.55, 5.15)	1.12 (0.546 2.76)	0.85 (0.30, 2.38)	0.36 (0.09, 1.39)	0.38 (0.08, 1.81)	2.21 (0.54, 9.04)	2.73 (0.56, 13.18)
Diastolic ≥85 mmHg	23 (15.3)	2.07 (0.69, 6.22)	2.20 (0.59, 8.16)	0.76 (0.29, 2.00)	0.46 (0.15, 1.41)	0.21 (0.05, 0.97)	0.16 (0.03, 0.90)	2.24 (0.47, 10.71)	3.50 (0.59, 20.68)

^*^Adjusted for age, sex, site location.

^**^Adjusted for age, sex, site location, marital status, education, paid employment, smoking, alcohol use, and fish intake.

**Table 6:** Adjusted* associations between metals (dichotomized at median) and cardiometabolic risk factors, controlling for percent body fat (%BF)

		Lead (>1.66 μg/dL)		Cadmium (>0.008 μg/L)		Arsenic (>8.48 μg/L)		Mercury (>1.49 μg/L)	
		Core model plus %BF	Fully-adjusted plus %BF	Core model plus %BF	Fully-adjusted plus %BF	Core model plus %BF	Fully-adjusted plus %BF	Core model plus %BF	Fully-adjusted plus %BF
Cardiometabolic risk factor	N (%)	OR (95% CI)*	OR (95% CI)**	OR (95% CI)*	OR (95% CI)**	OR (95% CI)*	OR (95% CI)**	OR (95% CI)*	OR (95% CI)**
Overweight, BMI ≥25	74 (49.3)	0.90 (0.34, 2.40)	0.88 (0.31, 2.51)	0.55 (0.23, 1.33)	0.44 (0.17, 1.15)	0.48 (0.12, 1.87)	0.36 (0.08, 1.61)	3.35 (0.82, 13.67)	3.24 (0.80, 13.12)
Obese, BMI ≥30	44 (29.3)	2.33 (0.73, 7.43)	2.70 (0.75, 9.75)	2.04 (0.78, 5.33)	1.99 (0.64, 6.12)	1.68 (0.37, 7.72)	1.43 (0.28, 7.23)	3.37 (0.67, 16.86)	4.18 (0.82, 21.31)
Waist Circumference ≥94 cm (males) or ≥80 cm (females)	74 (49.3)	2.23 (0.68, 7.24)	4.53 (1.06, 19.48)	1.09 (0.40, 2.95)	1.40 (0.45, 4.42)	1.19 (0.25, 5.60)	2.39 (0.38, 15.11)	2.91 (0.55, 15.40)	2.76 (0.52, 14.76)
Glucose ≥100 mg/dL (fasting)	51 (34.0)	4.82 (2.01, 11.53)	4.99 (1.97, 12.69)	2.01 (0.98, 4.13)	1.60 (0.72, 3.56)	4.81 (1.48, 15.58)	5.62 (1.49, 21.19)	2.12 (0.71, 6.33)	2.10 (0.66, 6.64)
LDL cholesterol ≥2.59 mmol/L	101 (67.3)	0.78 (0.35, 1.76)	0.77 (0.32, 1.86)	1.25 (0.60, 2.62)	0.75 (0.32, 1.76)	0.64 (0.21, 1.94)	0.50 (0.15, 1.71)	2.15 (0.73, 6.33)	1.78 (0.58, 5.51)
Triglycerides ≥1.7 mmol/L	45 (30.0)	0.12 (0.05, 0.32)	0.09 (0.03, 0.26)	0.38 (0.17, 0.83)	0.36 (0.15, 0.90)	0.63 (0.22, 1.84)	0.58 (0.18, 1.84)	1.37 (0.45, 4.14)	1.22 (0.38, 3.93)
HDL cholesterol <1.03 (males) or <1.29 (females) mmol/L	47 (31.3)	2.64 (1.08, 6.50)	2.10 (0.79, 5.61)	0.60 (0.29, 1.2)	0.61 (0.26, 1.44)	9.81 (2.41, 39.88)	8.02 (1.84, 35.01)	0.44 (0.14, 1.41)	0.50 (0.15, 1.63)
Triglyceride/HDL ratio >3.5 (males) or >2.5 (females)	55 (36.7)	0.14 (0.04, 0.43)	0.15 (0.04, 0.51)	0.42 (0.16, 1.13)	0.53 (0.18, 1.53)	0.51 (0.6, 1.62)	0.61 (0.17, 2.15)	1.31 (0.39, 4.36)	1.19 (0.32, 4.49)
C-reactive protein ≥10 mg/L	43 (28.7)	0.15 (0.06, 0.39)	0.13 (0.05, 0.38)	0.19 (0.08, 0.47)	0.27 (0.10, 0.73)	0.38 (0.12, 1.17)	0.50 (0.16, 1.62)	1.22 (0.39, 3.79)	1.14 (0.33, 3.96)
Systolic ≥130 mmHg	24 (16.0)	1.67 (0.60, 4.64)	1.77 (0.57, 5.45)	1.10 (0.45, 2.70)	0.80 (0.28, 2.28)	0.38 (0.10, 1.48)	0.38 (0.08, 1.82)	2.27 (0.55, 9.38)	2.79 (0.57, 13.68)
Diastolic ≥85 mmHg	23 (15.3)	2.35 (0.75, 7.37)	2.37 (0.62, 9.07)	0.74 (0.28, 1.96)	0.39 (0.12, 1.26)	0.22 (0.05, 1.00)	0.15 (0.03, 0.91)	2.30 (0.47, 11.15)	4.22 (0.67, 26.39)

^*^Adjusted for age, sex, site location, and percent body fat (%BF).

^**^Adjusted for age, sex, site location, marital status, education, paid employment, smoking, alcohol use, fish intake, and percent body fat (%BF).

## Discussion

These data, while not entirely consistent, are suggestive of potentially important associations between blood metals concentrations and cardiometabolic risk. In particular, a statistically significant relationship was observed between arsenic and lead exposure and increased risk for elevated fasting glucose which was stronger upon adjustment for adiposity. This is consistent with previously published cross-sectional studies examining these associations [28, 47, 48]. Additionally, arsenic was significantly associated with increased odds of low HDL cholesterol both with and without adjustment for percent body fat. Mercury was associated with increased odds for 10 of the 11 cardiometabolic risk factors though these associations were not statistically significant. We propose to further evaluate these preliminary associations in the larger METS cohort of 2,500 individuals at baseline and also assess changes in risk over time taking advantage of the longitudinal follow-up of the cohort.

There is increasing concern that unintended exposures to environmental contaminants may be adversely affecting human health. The association between environmental contaminants and diabetes, obesity, and cardiovascular disease is an emerging area of interest in the field of environmental health sciences [2, 49]. Despite the conventional focus on clinical and modifiable lifestyle risk factors, the incidence of cardiometabolic diseases continues to increase at alarming rates bringing the focus on environmental risk factors to the forefront. Simultaneously, there is increasing recognition that many chronic diseases have their origins in fetal life [50] and exposure to endocrine disrupting chemicals, such as metals, during critical windows of fetal development may be important contributing factors that “program” individuals to be at increased risk for metabolic disturbances by altering normal glycemic control or adipocyte differentiation [51, 52]. Nonetheless, important questions remain about timing of critical windows of exposure, susceptible populations at risk, and biological mechanisms of effect.

It is unclear whether adiposity may be a mediator, modifier, or outcome as a result of exposure to environmental chemicals, therefore, we presented the main outcome analyses with and without adjustment for adiposity. In most cases, the observed associations were stronger after adjustment for percent body fat. Adipose tissue is now recognized as an important endocrine organ responsible for maintaining homeostasis or making physiological adaptations in response to dietary lipids, including increased inflammation and insulin resistance [53]. Adipocytes secrete substances that contribute to peripheral insulin resistance, including adiponectin, resistin, TNF-alpha and interleukin 6 which interfere with glucose metabolism and exert lipotoxic effects on pancreatic beta cells [54]. However, most studies use BMI which cannot distinguish between overall lean vs. fat mass or body fat distribution. Future analyses in the larger METS cohort over time will be aimed at trying to disentangle these complex associations.

Our study has several strengths including the wide geographic distribution and resultant exposure variability in our subpopulations. By design, there is also a wide distribution of factors related to social and economic development and the subpopulations are relatively homogeneous with respect to ancestral/genetic background [39]. African descent is a known risk factor for cardiometabolic diseases [55] and this study will allow us to further elucidate the potential for gene-environment interactions in this high-risk subgroup.

Despite these strengths, our study has several limitations: the small sample size of this preliminary study does not allow us to rule out chance findings or fully explore the role of adiposity as a potential mediator or modifier of these relationships. There may be selection bias given the unequal distribution of risk factors, such as sex and BMI, in the randomly chosen subset of individuals at baseline. It may be that cumulative lifetime exposure to one or more metals, not adequately captured by blood levels measured cross-sectionally in our study, are responsible for adverse health effects of exposure. Finally, we did not control for dietary factors, other than fish intake, or measure speciated arsenic or mercury levels in these analyses.

The role of diet both in the absorption, distribution, and metabolism of metals and in relation to the outcomes of interest in this study cannot be underestimated. The sites in our parent study were chosen specifically to “model the epidemiologic transition” occurring across five diverse countries of varying levels of social and economic development. The changing dietary and physical activity patterns worldwide are responsible for the “double burden” of malnutrition and obesity in many developing countries [56]. Thus, when examining the role of environmental contaminants, micronutrient status may be an important modifier of the effects of exposure, particularly to metals [57–59].

Health disparities in environmental exposures have also been widely documented [60] and environmental and occupational health risks may disproportionately associated with social disparities, such as lower socioeconomic status, due to co-occurring risks such as poor housing conditions, employment conditions, and nutritional deficits. African American race has been associated with elevated blood lead levels independent of age and socioeconomic conditions [61, 62] and, in fact, social disadvantage may further modify or exacerbate the adverse effects of environmental exposures [63]. The concept of allostatic load suggests that the cumulative dysregulation of biological systems due to changing social and environmental stressors may result in decreased effectiveness of adaptive responses and increased risk for disease [64]. Genetic susceptibility to metals exposure is also recognized [65]. Divalent metal transporter-1 (DMT1) functions in the transport of some divalent metal ions across the plasma membrane [66, 67] and racial-ethnic differences in DMT1 expression may underlie racial disparities in blood metals levels [68].

Health disparities in endocrine diseases have recently been reviewed by the Endocrine Society which issued a scientific policy statement calling for multilevel interventions to reduce disparities in diabetes and endocrine diseases [69]. Our data suggest that exposure to environmental contaminants should be considered when evaluating risk and designing prevention and intervention programs. Racial disparities in health cannot be explained solely on the basis of genetic susceptibility, poverty and access to care, lifestyle or environmental factors [70]. While the size of the effects of exposures to environmental contaminants may be relatively small, their impact on a population-level is large, given the widespread and ever-changing distribution of environmental risk factors around the world and potential for cumulative effects from exposure to multiple pollutants simultaneously.

## Conclusions

These data are suggestive of potentially important associations between blood metals concentrations and cardiometabolic risk. Insights about potential biological mechanisms underlying disparities in cardiometabolic health can be gained from exploring differences in environmental exposures. Research efforts should include environmental factors when studying the complex etiology of cardiometabolic diseases which could result in targeted interventions to decrease health disparities and the associated risks of ongoing exposures particularly in the developing world where environmental and occupational control policies may be lacking or inadequate to protect public health.

## Abbreviations

BMI: Body mass index
DMT-1: Divalent metal transporter-1
HDL: High-density lipoprotein
HDI: Human Development Index
kg/m^2^: Kilogram per meter-squared
LOD: Limit of detection
LDL: Low-density lipoprotein
METS: Modeling the Epidemiologic Transition Study
OR: Odds ratio
C.I.: Confidence interval
NHANES: National Health and Nutrition Examination Survey
NIST: National Institute of Standard and Technology
SRM: Standard Reference Material
HNO_3_: Nitric acid
p: p-value
SD: Standard deviation
USA: United States of America.
